# Source Discrimination in Adults with Attention Deficit Hyperactivity Disorder

**DOI:** 10.1371/journal.pone.0065134

**Published:** 2013-05-31

**Authors:** Anselm B. M. Fuermaier, Lara Tucha, Janneke Koerts, Steffen Aschenbrenner, Matthias Weisbrod, Klaus W. Lange, Oliver Tucha

**Affiliations:** 1 Department of Clinical and Developmental Neuropsychology, University of Groningen, Groningen, The Netherlands; 2 Department of Clinical Psychology and Neuropsychology, SRH Clinic Karlsbad-Langensteinbach, Karlsbad-Langensteinbach, Germany; 3 Department of Psychiatry and Psychotherapy, SRH Clinic Karlsbad-Langensteinbach, Karlsbad-Langensteinbach, Germany; 4 Section for Experimental Psychopathology, Centre for Psychosocial Medicine, University of Heidelberg, Heidelberg, Germany; 5 Department of Experimental Psychology, University of Regensburg, Regensburg, Germany; University Of São Paulo, Brazil

## Abstract

**Objectives:**

The context of memory experiences is referred to as source memory and can be distinguished from the content of episodic item memory. Source memory represents a crucial part of biographic events and elaborate memory experiences. Whereas individuals with attention deficit hyperactivity disorder (ADHD) were shown to have inefficient item memory, little is known about the context of memory experiences.

**Methods:**

The present study compared 37 adult patients with a diagnosed ADHD with 40 matched healthy participants on a word list paradigm. Memory functions of encoding, retention and source discrimination were assessed. Furthermore, standardized measures of memory and executive control were applied in order to explore a qualitative differentiation of memory components.

**Results:**

Adult patients with ADHD showed impaired performance in encoding of new information whereas the retention of encoded items was found to be preserved. The most pronounced impairment of patients with ADHD was observed in source discrimination. Regression models of cognitive functions on memory components supported some qualitative differentiation.

**Conclusions:**

Data analysis suggests a differential pattern of memory impairment in adults suffering from ADHD with a particular deficit in source discrimination. Inefficient source discrimination in adults with ADHD can affect daily functioning by limiting biographic awareness and disturbing general cognitive processes.

## Introduction

Neuropsychological assessments revealed that adults with attention deficit hyperactivity disorder (ADHD) display impairments in various aspects of cognition [1], [2]. Because research put a lot of emphasis on executive dysfunction and inattention associated with ADHD, there is a considerable body of evidence showing that adults with ADHD are impaired with regard to working memory, inhibition, set shifting and planning as well as vigilance, selective attention and divided attention [3]–[8]. However, other aspects of cognition, such as memory functions, received less attention. Theoretical considerations implied that executive dysfunction may adversely affect memory functions of adults with ADHD. This is confirmed by the results of the meta-analyses performed by Hervey and colleagues [9] as well as Schoechlin and Engel [10] demonstrating that adults with ADHD suffer from disturbances of both verbal memory as well as figural memory as indicated by medium to small effects. Inefficient encoding and retrieval could repeatedly be shown in patients with ADHD, although retention of already learned information was found to be generally intact [11]–[14]. Studies on memory performance in ADHD primarily focused on episodic memory processes, including encoding, retention and retrieval of information. As primary measure, the number of correctly retrieved items was compared with the number of items which have been presented during a learning period. In this respect, studies focused on the content (but not on the context) of memory experiences.

In contrast to item memory in episodic remembering, the context of memory experiences, also referred to as *source memory*, has been widely neglected in research on ADHD. *Source memory* comprises all information about *where* and *when* the event took place and *how* information was acquired [15], [16]. For example, studies on memory functioning throughout lifespan showed that although older people have in general an intact memory about the facts of past events, information about when or where an event took place or where and from whom they learned certain facts, are less likely to be recollected with increasing age [17]. Detailed information about the source of events represents a crucial quality of human memory, since events of episodic memory become vivid and rich. Elaborated context information may also be responsible for an emotional connotation and personal evaluation of biographical events.

Previous research demonstrated that successful functioning in source memory requires cognitive processes which are associated with executive control, including verbal fluency and set shifting [15], [18], [19]. As impairments of executive control have reliably been observed in both children and adults with ADHD, one would expect that source memory is also impaired in these individuals [4], [20]–[22]. White and Marks [23] found a different pattern of source discrimination, a common paradigm to measure source memory, in undergraduate students showing characteristics of ADHD compared to students without these characteristics. Source memory judgments were not consistently poorer in students with characteristics of ADHD, but results differed between groups depending on how items have been encoded in the learning period. Despite the availability of source discrimination paradigms to measure source memory, and despite our knowledge about the associations between source memory and executive functioning as well as between ADHD and executive dysfunctioning, source discrimination has not been examined in patients diagnosed with ADHD.

Therefore, the aim of the present study was to assess source discrimination in adult patients with ADHD. The present study is the first to examine source memory in patients with ADHD by creating a word list paradigm integrating tasks of encoding, retention and source discrimination. Adults with ADHD were expected to show inefficient encoding of new information, although retention of already encoded material was hypothesized to be intact. Moreover, theoretically driven considerations supposed impaired abilities of adults with ADHD with regard to source discrimination. Finally, standard measures of cognition were applied and their contributions to memory components were explored in order to add conceptual clarity to the distinction of item memory and source memory.

## Methods

### Participants

Sixty-three adults with ADHD participated in the study. All adults with ADHD were outpatients, recruited from the Department of Psychiatry and Psychotherapy, SRH Group, Karlsbad-Langensteinbach, Germany. The diagnostic assessment was undertaken by experienced clinicians and involved a clinical psychiatric interview according to DSM-IV criteria for ADHD as devised by Barkley and Murphey [24] including the retrospective diagnosis of an ADHD in childhood (DSM-IV criteria) and current symptoms. Moreover, all participants completed two standardized self-report rating scales designed to quantify ADHD symptoms currently and retrospectively [25]. Childhood ADHD symptoms were self-rated with the short version of the Wender Utah Rating Scale (WURS-K) including 25 items on a five-point Likert scale [26]. Severity of ADHD symptoms in adulthood was self-rated with the ADHD Self-Report Scale consisting of 18 items on a four-point Likert scale corresponding to the diagnostic criteria of DSM-IV [25], [27]. Patients were selected according to age, diagnosis, intellectual functions (IQ), and willingness to participate in the study. Potential patients were excluded (I) if they had clinically significant chronic medical conditions, (II) if they were currently treated with any medication known to affect the central nervous system, (III) if there was a history suggestive of ‘psychosis’ (indicating schizophrenia, delusional disorder, depressive disorder with psychotic features or manic episode), (IV) if there was a history of neurological disorders including head injury, (V) if there was a history of substance abuse disorder during the previous two months, (VI) if the initial psychiatric assessment indicated a current major depressive episode, or (VII) if estimated premorbid verbal IQ was <85. Twenty-six of the 63 adults with ADHD were excluded (reasons for exclusion: current treatment with medication in n = 23; current psychotic symptoms in n = 2; history of neurological disorder in n = 1), resulting in a sample of 37 adults with ADHD. In the diagnostic assessment of the 37 patients with ADHD, 12 patients met DSM-IV criteria for ADHD – predominantly inattentive type (ADHD-I), 1 patient met criteria for ADHD – hyperactive-impulsive type (ADHD-H) and 24 patients met criteria for ADHD – combined type (ADHD-C). Eight of the 37 patients with ADHD were diagnosed with one or more comorbid psychiatric disorders, including mood disorders (n = 6), eating disorder (n = 1) and personality disorder (n = 1).

Furthermore, 40 healthy individuals were assessed. None of the healthy participants reported to have a history of neurological or psychiatric diseases and none were taken any medication known to affect the central nervous system at the day of the assessment. All healthy participants were recruited from the local community and completed the same self-report questionnaires for current and retrospective ADHD symptoms prior to the assessment [25]. Intellectual functions (i.e. vocabulary skills) of all participants were measured using the Multiple Choice Vocabulary Test [28]. Characteristics of patients with ADHD and healthy participants are presented in Table 1. Patients and healthy participants did not differ in age (t(75) = 0.46, p = .65), gender (χ^2^(1) = 0.19; p = .89) and intellectual functions (t(75) = 0.33, p = .74). As expected, healthy participants scored lower on both current and retrospective ADHD symptoms (t(75) = 12.83, p<.001 for current symptoms; t(75) = 12.51, p<.001 for retrospective symptoms).

**Table 1:** Characteristics of participants.

	Patients with ADHD (n = 37)	Control participants (n = 40)
Age (in years)	34.5±11.3	33.4±9.6
Gender (female/male)	21/19	20/17
Intellectual functions (IQ)^a^	100.4±11.9	101.2±8.3
WURS-K^b^	45.1±13.0	11.9±9.1
ADHD – Self-Report Scale	32.9±9.2	9.6±5.7

a Multiple Choice Vocabulary Test (MWT-B);

b Wender Utah Rating Scale – short version.

### Materials

#### Measurement of encoding, retention and source discrimination

An *Immediate Recognition Test* (encoding), a *Delayed Recognition Test* (retention) and a *Source Memory Test* (source discrimination) were designed using word lists.

The following materials were used for the word list paradigm: In total, five word lists consisting of unrelated German nouns were created. All words were drawn from the CELEX database using Wordgen v1.0 software toolbox [29]. All words were comparable in length (four to six letters), number of syllables (one or two) and frequency of use in German language. Four word lists containing 40 words each served as study lists (*List 1* and *List 2*) or distractor lists (*List 3* and *List 4*). To control for serial position effects (primacy and recency effects), five additional words were placed at the beginning and at the end of each study list. One study list and one distractor list were used in the *Immediate Recognition Test* (e.g. *List 1* and *List 3*), the remaining study list and distractor list were used in the *Delayed Recognition Test* (e.g. *List 2* and *List 4*). The use of study lists (*List 1* or *List 2*) in the study phase and distractor lists (*List 3* or *List 4*) in the recognition test was counterbalanced in both memory tests across participants in order to directly compare performance in immediate and delayed recognition tests. *List 5* was performed in the assessment of *source memory* and consisted of 28 words. For the presentation of the words in the *Source Memory Test*, *List 5* was split. Half of the words were displayed in blue font on the left hand side of a screen and the other half was presented in green font on the right hand side. This approach has been shown to be successful in measuring source information in previous studies [30], [31]. The allocation of words to be presented in blue font/left side or in green font/right side was counterbalanced across participants. To control for serial position effects (primacy and recency effects) in the *Source Memory Test*, three additional words were placed at the beginning and at the end of *List 5* at the time of presentation. An item recognition test was applied for all tests to keep requirements on effortful and organized retrieval strategies low and to focus on the processes of encoding and retention. Retrieval-induced forgetting represented a potential confounder [32], [33] and was controlled by retrieving one set of items not more than once. Therefore, the *Immediate Recognition Test*, *Delayed Recognition Test* and *Source Memory Test* were conducted independently for each participant by different set of words. The presentation of the words was computerized using E-Prime software 2.0.

*Encoding* was measured with the *Immediate Recognition Test.* In the study phase, all words (n = 40) from one study list (*List 1* or *List 2*) were presented consecutively in random order at the center of a screen (Arial, font size 44, screen size 15.4 inch). Each word appeared for four seconds on the screen before the next word was presented. Serial position effects (primacy and recency effects) were controlled for by placing five additional words at the beginning and the end of the study list. The participants were instructed to focus on the stimulus presentation and to use whatever mnemonics they thought were effective to memorize the words presented on the screen. A recognition test was performed immediately after the study phase. In the recognition test, all words from the study phase and one distractor list (*List 3* or *List 4*) were presented consecutively in random order at the center of the screen (80 words in total). Words used to control for primacy and recency effects were not presented in the recognition test. The participants were instructed to indicate with a button press on one of two predefined buttons on the keyboard whether the displayed word has been presented in the study phase or not. The test was self-paced and the next word appeared immediately after the participants gave a response. The number of correctly classified words was registered.

*Retention* was measured with the *Delayed Recognition Test.* In the study phase, all words (n = 40) from the study list which has not been presented in the *Immediate Recognition Test* (*List 1* or *List 2*) were presented consecutively in random order at the center of a screen (Arial, font size 44). Each word appeared for four seconds on the screen before the next word was presented. To control for serial position effects (primacy and recency effects), five words were placed at the beginning and at the end of the study list. Again, participants were instructed to focus on the stimulus presentation and to use whatever mnemonics they thought were effective to memorize the words presented on the screen. In the *Delayed Recognition Test*, a delay of 40 minutes followed the study phase. The participants were asked to perform some neuropsychological tests during the delay, including measures of short-term memory, working memory, flexibility, inhibition, verbal fluency, episodic retrospective memory and intellectual functions. After the delay, a recognition test was performed and all words from the study phase and the distractor list which have not been presented in the *Immediate Recognition Test* (*List 3* or *List 4*) were presented consecutively in random order at the center of the screen (80 words in total). Words used to control for primacy and recency effects were not presented in the recognition test. The participants were instructed to indicate with a button press on one of two predefined buttons on the keyboard whether the displayed word has been presented in the study phase or not. The test was self-paced and the next word appeared as soon as the participants gave a response. The number of correctly classified words was registered. Moreover, a measure of retention was obtained by calculating the quotient of the number of correctly classified words in the *Delayed Recognition Test* divided by the number of correctly classified words in the *Immediate Recognition Test.* Hence, the target measure of the *Delayed Recognition Test* was the percentage of correctly classified words in the delayed condition in relation to the immediate condition.

*Source discrimination* was measured with the *Source Memory Test.* Items in the *Source Memory Test* were presented in different color fonts (blue or green) and at different spatial locations (left or right side of the screen). In the study phase, all words of *List 5* were presented consecutively on a screen (Arial, font size 44). Half of the words (n = 14) were presented in blue font on the left hand side of the screen while the other half (n = 14) was presented in green font on the right hand side of the screen. The sequence of words was randomized. Each word appeared for seven seconds on the screen before the next word was presented. To control for serial position effects (primacy and recency effects) three words were placed at the beginning and the end of the list. The participants were instructed to focus on the stimulus presentation and to use whatever mnemonics they thought were effective to memorize the *words* AND the *corresponding source* of the words (blue font on the left side or green font on the right side). The source discrimination task was performed immediately after the study phase. All words of *List 5* were presented on the screen, displayed in black font at the center of the screen (Arial, font size 44). The participants were instructed to indicate *where/how* the word has been presented in the study phase, i.e. in blue font on the left side or in green font on the right side. The response was given by pressing one of two predefined buttons on the keyboard. The test was self-paced and the next word appeared as soon as the participants gave a response. The number of correctly classified words was registered.

#### Standard measures of cognition

*Short-term memory* was measured with the *Digit Span Forward task*, a subtest of the *Wechsler Memory Scale* [34]. Series of numbers were read to the participants who were required to repeat the digits in the same order as presented. The number of correctly repeated sequences was registered.

*Working memory* was measured with the *Digit Span Backward task*, a subtest of the *Wechsler Memory Scale* [34]. Series of numbers were read to the participants who were required to repeat the digits in the reversed order. The number of correctly repeated sequences was registered.

*Flexibility w*as measured with the *Trail Making Test* [35]. The *Trail Making Test* consisted of two parts. Part A required participants to draw a line, as fast as possible, between numbers in ascending order. Part B consisted of numbers and letters. Participants were required to switch attention between both concepts. They had to draw a line between both types of stimuli in ascending order, alternating between numbers and letters as fast as possible. The time in seconds to complete the test was registered. The target measure of the Trail Making Test for cognitive flexibility was the performance on part B (TMT-B).

*Inhibition* was measured with the *Stroop Color-Word Interference* task [36], [37]. The *Stroop Color-Word Interference* task consisted of three conditions. In the *Color Word* condition, 72 color words (YELLOW, GREEN, BLUE and RED) printed in black ink were presented on a card and participants were required to read them in clear voice as fast as possible. In the *Color Block* condition, 72 colored rectangles (rectangles printed in yellow, green, blue and red) were presented on a card and participants were required to name the color of the rectangles as fast as possible. In the *Color-Word Interference* condition, 72 color words (YELLOW, GREEN, BLUE and RED) were presented and printed in mismatching ink (e.g. RED printed in blue ink). The participants were required to name the color of the ink as fast as possible and to ignore the meaning of the printed word. The time in seconds to complete each trial was registered. A measure of inhibition was calculated for each participant by subtracting the time needed for completion of the *Color Block* condition from the time needed for the *Color-Word Interference* condition [4].

A test for *Verbal fluency* was applied (S-Word Test) which is similar to the Controlled Oral Word Association Test [38]. Participants were asked to produce, within 2 minutes, as many different words as possible beginning with the letter “S”. Names (e.g. “Steve, Stockholm, Sweden”), words beginning with another letter, nonexistent or foreign language expressions, words with the same stem (e.g. “sport, sport ground, sport badge”) and perseverations of words already given as a response were regarded as rule violations [39]. The number of correctly produced words was registered.

*Episodic retrospective memory* was assessed by the *Logical Memory test*, a subtest of the *Wechsler Memory Scale* [34]. Two short stories were read to the participants who had to recall the stories immediately after the presentation. The number of correctly recalled items was registered as a measure of *immediate recall*.

*Intellectual functions (i.e. vocabulary skills)* were measured using the Multiple Choice Vocabulary Test [28]. This test consists of 37 lines, each comprising of one authentic word and four fictitious words. The participants were required to find the authentic word by underlining it. The Multiple Choice Vocabulary Test is a valid and short test procedure which assesses vocabulary skills as a measure of intellectual functioning.

### Procedure

All participants were tested individually. Participants gave written informed consent to participate in the study at the beginning of the experiment. Subsequently, the memory paradigms were conducted. The word list paradigms were divided in three parts: The *Immediate Recognition Test*, the *Delayed Recognition Test* and the *Source Memory Test*. The order of immediate and delayed recognition tests was counterbalanced across participants in order to control for learning and interference effects. During the 40-minutes delay of the *Delayed Recognition Test*, standard measures of cognition were applied. The *Source Memory Test* was placed at the end of the procedure for all participants. All participants were debriefed at the end of the assessment. The total duration of the assessment was about 70 minutes.

### Ethics Statement

The study was conducted in compliance with the Helsinki Declaration. Ethical approval was obtained by the ethics committee of the medical faculty of the University of Heidelberg, Germany. All participants gave written informed consent prior to the assessment.

### Statistical Analysis

Multivariate analysis of variance (MANOVA) was applied to compare the performance of patients with ADHD and healthy participants on cognitive tasks. Effect sizes (η^2^, Cohen’s d) were calculated for all comparisons. The index η^2^ provides information about the proportion of variance which is accounted for by the factor group membership. As described by Cohen [40], η^2^ is a function of the effect size index f. According to Cohen [40], a small effect size (f = .10) corresponds to an η^2^ = .0099, a medium effect size (f = .25) to an η^2^ = .0588 and a large effect size (f = .40) to an η^2^ = .1379. For pairwise comparisons of means, negligible effects (d <0.20), small effects (d = 0.20), medium effects (d = 0.50) and large effects (d = 0.80) were distinguished [40]. Furthermore, Pearson product-moment correlations were applied separately for patients and healthy participants to test for significant relationships between memory paradigms of encoding, retention and source discrimination. With respect to correlation analyses, negligible effects (r <0.1), small effects (r = 0.1), medium effects (r = 0.3) and large effects (r = 0.5) were distinguished [40]. Moreover, the contribution of standard measures of cognition to memory functions of encoding, retention and source discrimination were estimated separately by using multiple regression analyses (method: forced entry (“enter”)). To maximize statistical power and to allow a common metric by which patients with ADHD and healthy participants are analyzed, all participants were included (n = 77). A significance level of α = .05 was set for all tests. Data analysis was performed using SPSS 18 for Windows.

## Results

### Group Differences in Cognitive Functions

**Figure 1:**
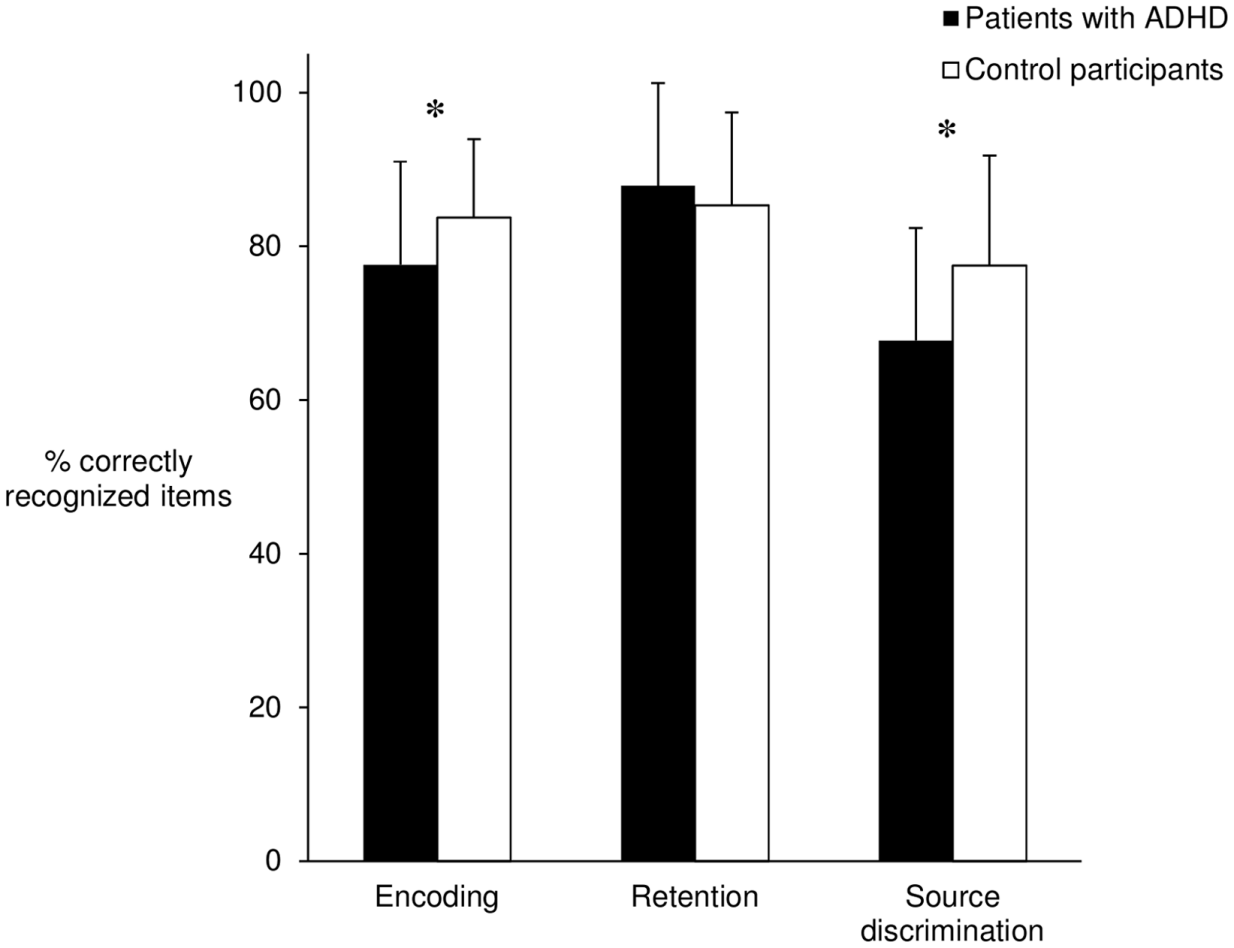
Encoding, retention and source discrimination of patients with ADHD and control participants (M±SD). Note: Data transformed; **Encoding**: Percentage of correctly recognized items in the immediate recognition test; **Retention**: Percentage of correctly recognized items in the delayed recognition test compared to the correctly recognized items in the immediate recognition test; **Source discrimination**: Percentage of correctly recognized items in the source memory test; * Significant at p<.05.

As indicated by a medium significant effect, patients with ADHD and healthy participants differed with regard to their performance in the experimental memory tasks (MANOVA: Wilk’s lambda = 0.890, F(3,73) = 3.001, p<.001, η^2^ = .110). Subsequent data analysis revealed that patients with ADHD showed a significantly decreased performance in the encoding of information (F(1,75) = 5.250, p = .025, d = 0.53) and in source discrimination (F(1,75) = 8.867, p = .004, d = 0.68). Both effects were of medium size. No significant difference was observed for retention of already encoded material (F(1,75) = 0.793, p = .376, d = 0.20) (Table 2). Group differences in experimental memory paradigms are presented in Figure 1. Data were transformed and are shown as percentage of correctly recognized items for all measures.

**Table 2:** Group differences in cognitive performance between patients with ADHD (n = 37) and control participants (n = 40).

	Patients with ADHD	Control participants	p	ES^a^
**Experimental memory paradigms**				
Encoding^b^	62.0±10.7	67.0±8.2	.025*	0.53
Retention^c^	87.9±13.3	85.3±12.1	.376	0.20
Source discrimination^d^	18.9±4.1	21.7±4.0	.004*	0.68
**Standard measures of cognition**				
Short-term memory^e^	6.8±1.7	7.7±2.0	.032*	0.50
Working memory^f^	6.3±1.9	6.7±2.0	.466	0.17
Flexibility^g^	70.8±23.6	60.8±20.2	.050*	0.46
Inhibition^h^	39.0±16.9	26.9±10.6	<.001*	0.87
Verbal fluency^i^	21.0±6.1	24.7±8.0	.029*	0.52
Retrospective memory^j^	23.5±6.9	30.9±6.2	<.001*	1.14

a Effect sizes indicated by Cohen’s d;

b Number of correctly recognized items in the immediate recognition test;

c Percentage of correctly recognized items in the delayed recognition test divided by the correctly recognized items in the immediate recognition test;

d Number of correctly recognized items in the source memory test;

e Digit Span Forward task (number of correctly repeated sequences);

f Digit Span Backward task (number of correctly repeated sequences);

g Trail Making Test part B (TMT-B) (time in seconds);

h Stroop Color-Word Interference task (time (in seconds) needed for the *Color-Word Interference* condition – time (in seconds) needed for the *Color Block* condition);

i Word Fluency Test (S-Word Test) (number of correctly produced words);

j Logical Memory from the Wechsler Memory Scale (number of correctly recalled items);

* Significant at p≤.05.

Further analysis indicated a large difference between patients and healthy participants in standard measures of cognition (Wilk’s lambda = 0.657, F(6,70) = 6.081, p<.001, η^2^ = .343). Compared to healthy participants, patients with ADHD showed a significantly decreased performance on all tests except of a negligible difference in working memory. Significant group differences in cognitive functioning ranged from small to large size (Table 2).

### Multiple Correlation Analysis

With regard to the group of healthy participants, correlation analyses between experimental measures of memory functions revealed a large significant relationship between encoding and source discrimination (r = .68; p<.001). Non-significant small correlations were observed between encoding and retention (r = −.15; p = .35) and between retention and source discrimination (r = .21; p<.20). With regard to the group of patients, data analysis revealed significant correlations for all three relationships (encoding and retention: r = −.63; p<.001; encoding and source discrimination: r = .68; p<.001; retention and source discrimination r = −.39; p = .018). Correlations were of medium to large size.

Multiple regression analyses were performed to examine a qualitative distinction between memory components (Table 3). A significant regression model explaining 34.8% of the total variance was found for encoding (F(6,70) = 6.24; p<.001). In this model, retrospective memory and verbal fluency accounted for a significant proportion of variance in encoding new information. Retrospective memory was found to have best predictive power explaining alone 25.2% of the total variance (r = .502), whereas verbal fluency alone explained 19.2% (r = .438) of the total variance. Both predictors positively affected encoding in the word list paradigm, such as that a higher performance in retrospective memory and verbal fluency resulted in an increased encoding of new information. Moreover, a significant regression model was obtained for the performance in source discrimination (F(6,70) = 4.81; p<.001) explaining 29.2% of the total variance. In this model, only verbal fluency contributed significantly to participants’ performance in source discrimination by explaining 20.5% of the total variance (r = .453). The association was positive indicating that higher verbal fluency performance resulted in better performance with regard to source discrimination. In contrast, no significant regression model was found for retention (F(6,70) = 1.60 p = .160). None of the cognitive functions assessed contributed significantly to the retention of already learned information.

**Table 3:** Summary of multiple regression models (method: forced entry („enter“)) for predicting encoding, retention and source discrimination.

Predictor variables	B	SE B	β	t	p
**Encoding**					
Short-term memory^a^	0.04	0.66	0.01	0.06	.955
Working memory^b^	0.16	0.59	0.03	0.28	.784
Flexibility^c^	−0.05	0.05	−0.11	−1.03	.305
Inhibition^d^	−0.02	0.07	−0.03	−3.2	.748
Verbal fluency^e^	0.37	0.14	0.28	2.54	.013*
Retrospective memory^f^	0.46	0.15	0.35	3.11	.003*
Total R^2^ = 34.8*					
**Retention**					
Short-term memory^a^	1.52	0.99	0.23	1.53	.130
Working memory^b^	−1.11	0.89	−0.17	−1.25	.217
Flexibility^c^	0.14	0.07	0.25	1.95	.055
Inhibition^d^	−0.12	0.10	−0.14	−1.12	.268
Verbal fluency^e^	−0.15	0.22	−0.09	−0.67	.503
Retrospective memory^f^	−0.21	0.22	−0.12	−0.94	.350
Total R^2^ = 12.1					
**Source discrimination**					
Short-term memory^a^	0.25	0.30	0.11	0.83	.412
Working memory^b^	0.23	0.27	0.10	0.85	.400
Flexibility^c^	0.01	0.02	0.06	0.51	.612
Inhibition^d^	−0.02	0.03	−0.07	−0.65	.517
Verbal fluency^e^	0.19	0.07	0.33	2.91	.005*
Retrospective memory^f^	0.19	0.07	0.18	1.54	.129
Total R^2^ = 29.2*					

a Digit Span Forward task;

b Digit Span Backward task;

c Trail Making Test part B (TMT-B);

d Stroop Color-Word Interference task;

e Word Fluency Test (S-Word Test);

f Logical Memory test from the Wechsler Memory Scale*;*

* Significant at p<.05.

## Discussion

### Effects on Encoding and Retention

In the present study, item memory and source memory were assessed by applying an integrated paradigm on adults with ADHD. Patients showed inefficient encoding of item information as measured in the immediate recognition test. Cognitive processes in the immediate recognition test can be attributed primarily to demands of encoding as it was not asked for long retention. Furthermore, by cuing the responses in a recognition test, the paradigm performed did not require complex retrieval strategies. In contrast, no significant difference was observed between patients and healthy participants in the forgetting rate of learned information as measured in the delayed recognition test. The present results therefore indicate that patients with ADHD have intact abilities in retention once information is successfully encoded and stored in memory. Results concerning encoding and retention were in accordance with our expectations as memory impairments in adults with ADHD were hypothesized only in those domains with high executive load. The role of executive functions in memory processes were emphasized by several studies on individuals with ADHD. Dysexecutive functions were found to be highly related to impaired prospective memory in adults with ADHD [41] and intact executive functions were attributed to efficient encoding and retrieval processes [11]–[14]. Individuals with ADHD were found to be highly susceptible in those executive operations required in encoding and retrieval, including semantic clustering, effortful rehearsal, strategic use of effective mnemonics and careful consideration of response alternatives [13]. However, retention of learned information does not primarily depend on these cognitive processes. In the treatment of cognitive impairments of adults with ADHD, it is therefore reasonable to teach how to strategically organize material for successful storage in memory. Furthermore, adults with ADHD could benefit from being taught how to make use of effective retrieval strategies when information is recollected from memory.

### Effects on Source Discrimination

Source memory can be qualitatively distinguished from item memory and represents an important part of human episodic memory containing crucial information of autobiographic events. The present study is the first to reveal decreased performance in source discrimination in patients with ADHD compared to healthy individuals. A different pattern of source discrimination has been demonstrated in students with characteristics of ADHD [23], however, a clinical sample of individuals diagnosed with ADHD has not yet been assessed. Consequences of impairments in source discrimination can be crucial, as biographic events become vivid and rich by detailed contextual information and past episodes are appreciated by elaborate context information. Losing context information (about the *where* and *when* of past episodes) may cause the recollection of such events meaningless as it is the source information that attributes an event its unique signature. For example, *flashbulb memories* represent vivid and enduring memory recollections about the circumstances of how one learned about surprising and emotionally relevant events (the reception of the event) [42]. In this respect, Davidson and colleagues [43] examined memory for the tragic September 11^th^, 2001, disaster. The authors showed selective deficits in patients with frontal lobe lesions about the reception of the event (*flashbulb memories*), although their memory for the target event was unimpaired. Deficient source memory in patients with ADHD may therefore negatively affect flashbulb memories. Furthermore, deficient source memory could be shown to be associated with general cognitive impairments, including increased interference in working memory, false recognition, cryptomnesia (a memory bias whereby a forgotten event returns without it being recognized as such) and overreliance on stereotypes during recollection [44]. In conclusion, inefficient source discrimination in individuals with ADHD may lead to a poverty of memory experiences of autobiographic episodic events and is related to general cognitive impairments which are crucial for everyday life.

Analysis of patients’ performances revealed significant correlations of medium to large size between encoding, retention and source discrimination, suggesting interrelated memory components rather than three qualitative independent components. In healthy participants, however, not all three memory components were interrelated as indicated by non-significant associations between retention and both source discrimination and encoding. In healthy adults, a significant relationship was only found between encoding and source discrimination which appears reasonable considering the high demand of the source memory task with regard to the encoding of source information. The differences between adults with ADHD and healthy adults concerning the relationships between memory components might have resulted from the impact of a moderator variable, such as a general distractibility or increased impulsivity in patients with ADHD which might have affected cognition in general. Consequently, test scores on a variety of cognitive tasks appear interrelated. The qualitative differentiation between item and source memory is supported by the results of multiple regression analyses of the present data. Performance in source discrimination was not predicted by retrospective memory (13.8% explained variance) but was shown to be significantly predicted by verbal fluency (20.5% explained variance), a common measure for divergent thinking associated with executive functions [5]. Other measures of executive functions did not considerably contribute to source discrimination. This lack of significant correlations is consistent with previous reports and has been explained by high inter- and intra-subject variability among patients as well as with the possibility that standard tests of executive functions measure a variety of different processes and may consequently depend partly on non-executive components [43], [45]. In contrast to the results regarding source discrimination, encoding new information was significantly predicted by episodic retrospective memory which explained 25.2% of the total variance. Verbal fluency was also found to significantly contribute to performance in encoding (19.2% explained variance), although predictive power was smaller. In accordance to our expectations, source discrimination was primarily explained by a measure of executive functions (i.e. verbal fluency), whereas encoding item information could be best explained by a measure of retrospective memory. No significant model was found to predict retention of encoded information which underlines the notion that retention as assessed by a recognition paradigm might not be primarily associated with measures of executive functions [11]–[14].

On the basis of several studies, Glisky and colleagues [17] assumed that deficient encoding processes in older adults are the most likely reason for inefficient source discrimination. However, as a methodological limitation in many studies comparing item memory with source memory, participants have been instructed to memorize item information whereas the source discrimination tasks applied in these studies were not explicitly mentioned to the participants [19], [46]. Consequently, performance in encoding source relevant information could have been enhanced in these studies by introducing task-orienting cues which directly address the relevance of memorizing source information [17]. With regard to the present study on adults with ADHD, participants’ item and source memory can directly be compared as both item information and source information have been explicitly mentioned in the instructions. The present results therefore support the conclusion of impaired encoding as the most likely reason for deficient source memory as no free recall with high demands on retrieval strategies was required and because performance in encoding and source discrimination were highly correlated in both samples assessed.

In conclusion, encoding, retention and source discrimination were assessed in an integrated design in adult patients with ADHD. Adults with ADHD showed an impaired encoding of new information whereas retention of learned material appeared to be intact. Most importantly, the largest effect was found for inefficient source discrimination which might adversely affect both the generation of elaborate and detailed contextual information about biographic events and the general cognitive efficiency of patients with ADHD.

### Limitations and Future Directions

In the present study, a word list paradigm was designed in order to distinguish between item memory and source memory. Some qualitative differentiation between these two concepts could be supported by regression analyses. However, it needs to be considered that there is a great similarity between both memory concepts which limits a qualitative differentiation. Demands of encoding, storing and retrieving information are present in tasks of both item memory and source memory. Data analysis emphasized a substantial overlap as shown by significant correlations between encoding, retention and source discrimination and therefore the segregation between item memory and source memory might not be fully justified.

Moreover, the straightforward approach to operationalize source information (information presented in different spatial locations and in different color fonts) might appear oversimplified. The spatial location on the screen (the “where” information) was redundant with the color font (the “how” information). Furthermore, it was not asked for “when” information has been presented. Hence, even though the present paradigm assessed crucial characteristics of source memory, the complex nature of source memory may not be fully captured in the present study.

In order to indicate the magnitude of group differences in encoding, retention and source discrimination, effect sizes were calculated for these measures. However, these values are not directly comparable as they depend on the difficulty of the individual tasks. Therefore, it would be of interest to assess encoding, retention and source discrimination repeatedly in tasks of various difficulties (by using different list sizes and time delays) in order to obtain the maximum impairment of each measure which can be compared to impairments in other measures.

Finally, the present study is the first to show impaired source discrimination in adults with ADHD and therefore requires replication in future research, preferable by increasing the sample size. It would also be of interest to examine group differences in source discrimination among subtypes of ADHD (inattentive subtype, hyperactive-impulsive subtype, combined subtype) in order to determine whether a deficit in source discrimination is characteristic only for a subgroup of patients with ADHD. An explorative analysis of the present data did not reveal a significant difference in source discrimination between patients of the inattentive subtype and patients of the combined subtype (data not shown). However, sample sizes of patients of the same subtype were small and therefore neither allow a reliable analysis nor conclusion.
